# Myasthenia Gravis Associated With COVID-19 Infection

**DOI:** 10.7759/cureus.39506

**Published:** 2023-05-25

**Authors:** Waleed Sadiq, Madeeha Subhan Waleed, Taqi A Rizvi, Shahkar Khan, Halim El Hage

**Affiliations:** 1 Pulmonary and Critical Care Medicine, Staten Island University Hospital, Staten Island, USA; 2 Internal Medicine, Lower Bucks Hospital, Bristol, USA; 3 Internal Medicine, Staten Island University Hospital, Staten Island, USA

**Keywords:** covid-19, autoimmune, sars-cov-2, sars, coronavirus disease, coronavirus, myasthenia gravis (mg)

## Abstract

COVID-19 first emerged in Wuhan, China in late December 2019. The disease majorly involves the lungs leading to various respiratory complications; however, neurological manifestations of the disease are also described in the literature. Here, we report a case of COVID-19-induced seronegative myasthenia gravis (MG). We discuss the cases of COVID-19 and MG already described in the literature in regard to their presentation and serological findings to better understand the association between the two disease processes. MG may be missed in patients after COVID-19 infections because of the comorbidities and anti-acetylcholine receptor and anti-muscle-specific tyrosine kinase antibodies being negative. Evidence from more studies will help analyze the pathological timeline of the disease process and the immunological characteristics of COVID-19-induced MG which can prove to have morbidity and mortality benefit in patients with COVID-19-induced MG.

## Introduction

COVID-19 emerged in Wuhan, China in December 2019. Major manifestations of COVID-19 are respiratory complications; however, neurological manifestations of the disease have also emerged over time [1]. Myasthenia gravis (MG) is an autoimmune condition with autoantibodies against the nicotinic acetylcholine receptors located at the neuromuscular junction [2]. Patients between the ages of 21 and 65 have been diagnosed with new-onset MG with positive anti-acetylcholine receptor (AChR) autoantibodies post-COVID-19 infection [3-5]. Moreover, cases of patients with anti-muscle-specific tyrosine kinase (MuSK)-MG have also been reported after COVID-19 infection [6].

Here, we report a case of COVID-19-induced seronegative MG with a review of cases already described to understand the association between the two disease processes.

## Case presentation

**Figure 1:**
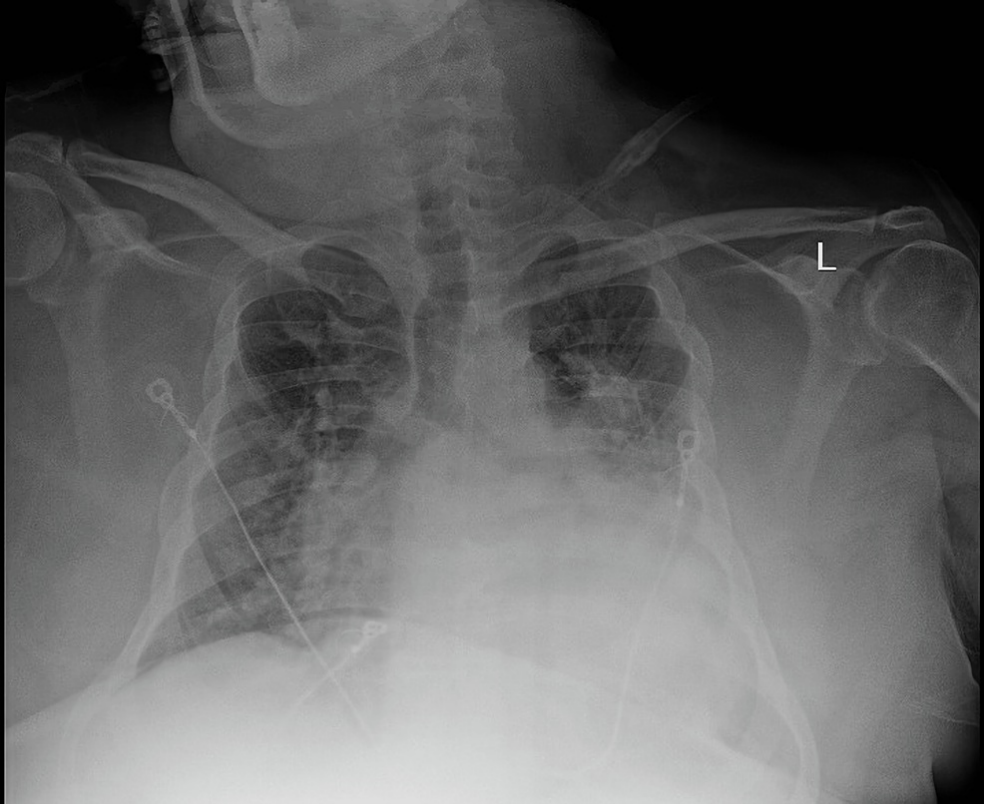
Chest X-ray showing bilateral opacities left greater than right.

A 46-year-old male with a past medical history of hypertension, hyperlipidemia, morbid obesity (body mass index of 38 kg/m^2^), obstructive sleep apnea, pes excavatum, interstitial lung disease, and recent hospitalization five months earlier due to acute hypoxic respiratory failure secondary to COVID-19 infection requiring mechanical ventilation presented to the emergency department with worsening shortness of breath that started a few days ago. He stated that he had recent exposure to sick children and had developed shortness of breath and a dry cough for a few days. However, his viral panel and COVID-19 testing were negative on admission. His vitals on presentation were as follows: his blood pressure was 128/82 mmHg, heart rate was 93 beats per minute, the temperature was 99.7°F, respiratory rate was 26 breaths per minute, and oxygen saturation was 91% on 3 L of oxygen via a nasal cannula. A Chest X-ray showed bilateral opacities left greater than right (Figure 1).

His pertinent laboratory values are listed in Table 1.

**Table 1:** Pertinent lab findings.

Laboratory parameters	Value	Reference range
White blood cell count	10.34 K/µL	4.80–10.80 K/µL
Hemoglobin	13.9 g/dL	14.0–18.0 g/dL
Platelet count	200 K/µL	130–400 k/µL
The partial pressure of carbon dioxide (arterial)	99 mmHg	42–55 mmHg
pH	7.27	7.32–7.43
Oxygen (arterial)	55 mmHg	80–100 mmHg
Serum bicarbonate	42 mmol/L	17–32 mmol/L
Anti-low‐density lipoprotein receptor-related protein 4 antibody	Positive	Positive/negative

Shortly after presenting to the emergency department, the patient’s respiratory status worsened, and he was placed in non-invasive ventilation, despite which he showed no improvement. The arterial blood gas showed a worsening pH of 7.20, partial pressure of carbon dioxide of 127 mmHg, and his mental status started to decline. He was intubated shortly after and started on intravenous ceftriaxone and azithromycin for suspected bacterial pneumonia.

Over the following days, the critical care team found it difficult to wean the patient off mechanical ventilation as he continued to fail spontaneous breathing trials. He was also found to have proximal muscle weakness along with ptosis, so neurology was consulted for further evaluation. Anti-AChR autoantibodies and anti-MuSK-MG were negative. However, antibodies against lipoprotein-related protein 4 were positive suggesting double-seronegative MG. He was started on pyridostigmine 60 mg TID with good response and was extubated consequently. The patient underwent a CT scan which was negative for a thymoma.

## Discussion

MG is a disease that causes localized or generalized muscle weakness due to antibodies not only against AChR and MuSK but other AchR-related proteins as well, which are located in the post-synaptic muscle membrane [7].

Infectious insults can trigger new-onset MG and other autoimmune diseases and cause disease deterioration [8]. SARS-CoV-2-induced autoimmune diseases including MG have been described in the literature [9]. COVID-19 seems to show an affinity for neural tissue as there have been reports of encephalitis, encephalopathy, cranial neuropathies, Guillain-Barré syndrome, rhabdomyolysis, anosmia, and ageusia [1]. Although the pathophysiology of COVID-19-induced MG has not been established yet, there are a few postulated mechanisms. These mechanisms include molecular mimicry as some COVID-19 proteins may be similar to human antigens [3-5,10-13] and an increased release of type 1 interferon and other inflammatory cytokines 14. Other hypothesized mechanisms include a breakdown of self-tolerance mechanisms after infection [11,12,14,15] and activation of latent MG 1 [4,15]. There have been a few cases of MG after COVID-19 [3-5,10-17], but to our knowledge, seronegative MG post-COVID-19 has not been described yet. It is important to recognize that seronegative MG can occur after COVID-19 as a delay in diagnosis can possibly worsen outcomes.

It is important to remember our patient received azithromycin which is not recommended in patients with underlying MG as it can worsen their condition [18]. This information coupled with the fact that our patient had a latency of 152 days between infection and MG onset raises the question of whether azithromycin worsened the patient’s subclinical, undiagnosed MG.

The details of the cases we reviewed are mentioned in Table 2.

**Table 2:** Cases of myasthenia gravis associated with COVID-19 described in the literature. anti-AChR: anti-acetylcholine receptor; Abs: antibodies; MG: myasthenia gravis; RNS: repetitive nerve stimulation; SFEMG: single fiber electromyography; LRP4: lipoprotein-related protein 4; MuSK: muscle-specific tyrosine kinase; MCD: mean consecutive differences

Author	Gender	Age	Days for myasthenia to present post-COVID-19	Repetitive stimulation test	Antibodies	Myasthenia type	Outcomes
Restivo et al., 2020 [4]	Male	64	5	Repetitive stimulation of the facial nerve showed a 57% decrement	Anti-AChR Abs +	Generalized MG	Responsive to pyridostigmine bromide and prednisone
	Male	71	5	Ulnar RNS 56% decrement	Anti-AChR Abs +	Generalized MG	Improved with plasmapheresis
	Male	68	7	RNS showed facial (52%) and ulnar (21%) nerve decrement	Anti-AChR Abs +	Generalized MG	Responsive to one cycle of intravenous immunoglobulin treatment
Pérez Álvarez et al., 2020 [13]	Male	48	15	Not done	Anti-AChR Abs +	Ocular MG	The patient improved with hydroxychloroquine and azithromycin
Assini et al., 2021 [14]	Male	77	56	RNS and SFEMG consistent with MG	Anti-AChR Abs−; anti-MuSK Abs +; anti-LRP4 Abs−	Oculobulbar MG	Pyridostigmine (60 mg four times a day) with unsatisfactory clinical response, followed by immunosuppressive therapy (azathioprine 1.5 mg/kg/day) with an improvement in MG
Huber et al., 2020 [3]	Female	21	10	Facial RNS was normal. SFEMG not performed	Anti-AChR Abs +; anti-MuSK Abs−; anti-LRP4 Abs−; anti-titin Abs−	Ocular MG	Intravenous immunoglobulins and oral pyridostigmine.
Muhammed et al. 2021 [15]	Female	24	28	Ulnar RNS and facial SFEMG consistent with MG	Anti-MuSK Abs +	Generalized MG	Improved with intravenous immunoglobulin, pyridostigmine, and prednisolone
Sriwastava et al ., 2021 [5]	Female	65	11	Facial RNS and SFEMG consistent with MG decremental response over more than 10% on RNS of orbicularis oculi	Anti-AChR Abs +; anti-MuSK Abs−	Ocular MG	Pyridostigmine 60 mg every six hours
Karimi et al., 2021 [10]	Female	61	32	Facial, median, and accessory nerve RNS showed a 15–27% decrement. SFEMG not performed	Anti-AChR Abs +	Generalized MG	Plasma exchange, pyridostigmine bromide 60 mg four times a day, and prednisone 1 mg/kg with significant improvement
	Male	57	6	Facial, ulnar, median, and radial RNS showed a 10–40% decrement. SFEMG not performed	Anti-AChR Abs +	Generalized MG	Pyridostigmine 60 mg three times a day and prednisone 25 mg daily
	Female	38	28	Facial and radial RNS showed a 30–40% decrement SFEMG not performed	Anti-AChR Abs +; anti-MuSK Abs−	Generalized MG	Pyridostigmine 240 mg and prednisolone 25 mg daily
Muralidhar et al ., 2021 [12]	Male	65 years	42	Facial RNS showed a 41% decrement; accessory nerve RNS showed an 18.1% decrement	Anti-AChR Abs +; anti-MuSK Abs −	Generalized MG	Intravenous immunoglobulin, prednisolone, and pyridostigmine
Tereshko et al ., 2022 [17]	Female	19	13	Facial RNS showed 60% decrement; ulnar RNS showed 37% decrement; SFEMG frontalis muscle MCD = 103 μs	Anti-AChR Abs +; anti-MuSK Abs −	Oculobulbar and then generalized MG	Oral pyridostigmine 60 mg QID, oral pyridostigmine extended-release 180 mg, prednisone 50 mg, and intravenous immunoglobulin during readmission
Jõgi et al., 2022 [11]	Male	65	14	Facial RNS showed >50% decrement, ulnar RNS showed 5–10% decrement, and accessory nerve RNS showed 26–29% decrement. SFEMG not performed	Anti-AChR Abs +; anti-titin Abs +	Generalized MG	Prednisolone and a five-day course of intravenous immunoglobulin for a total dose of 180 g
Taheri et al., 2022 [16]	Female	35	27	SFEMG consistent with MG; RNS not performed	Anti-AChR Abs +	Generalized MG	Pyridostigmine 60 mg three times a day for as long as the patient remained symptomatic
Bhandarwar et al., 2021 [19]	Male	61	60	NA	Anti-AchR Abs+	Generalized MG	Oral prednisolone 30 mg
Current report	Male	46	152	Not performed	Anti LRP4 Abs+	Generalized MG	Pyridostigmine 60 TID

In summary, 13 patients had anti-AChR antibodies, two patients had anti-MuSK antibodies, and one had both anti-AChR antibodies and anti-titin antibodies. The average age of diagnosis was 51.8 [19], and the average time to onset of symptoms post-COVID-19 infections was 19.9 days [5]. MG manifested as generalized muscle weakness in 10 patients, whereas three patients presented with ocular MG. Two patients had an oculobulbar presentation; one of the patients then progressed to generalized muscle weakness. Five patients initially needed at least one course of intravenous immunoglobulin (IVIg), and two patients underwent plasmapheresis. Twelve patients were treated with pyridostigmine and/or prednisone as initial therapy or after IVIg/plasmapheresis and three patients needed azathioprine in addition. All patients were reported to have improved after therapy. For one case, the therapy was not described [11]. A prospective follow-up of patients with post-COVID-19 MG will provide valuable information about their prognosis and outcomes which could provide clarity on the association between COVID-19 and other autoimmune conditions. MG may present in elderly people; however, the diagnosis can be missed because it may go unrecognized due to the comorbidities and when patients are seronegative on the standard radioimmunoassay and immunohistochemistry.

## Conclusions

To our knowledge, this is the first reported case of double-seronegative MG post-COVID-19 infection. The mechanisms for the development of MG post-COVID-19 are unknown. MG may be missed in patients after COVID-19 infections because of the comorbidities and anti-AChR and anti-MuSK antibodies being negative. This article raises awareness regarding the association between the two conditions; however, evidence from more case series is important to analyze the pathological timeline and the immunological characteristics of COVID-19-induced MG which can prove to have morbidity and mortality benefit in patients, especially with respiratory compromise.
